# A 70 year old male with difficulty in breathing

**DOI:** 10.4103/1817-1737.49417

**Published:** 2009

**Authors:** Sarita Magu, Shalini Agarwal, Sanjay Kumar

**Affiliations:** *Department of Padiology, Pt. BD Sharma, Post Graduate Institute of Medical Sciences, Rohtak (Haryana) India*; 1 *Department of Pathology, Pt. BD Sharma, Post Graduate Institute of Medical Sciences, Rohtak (Haryana) India*

A 70 year old man presented with complaints of moderate pain chest which was generalized in nature & difficulty in breathing for the past 6-7 month. He also had cough off and on. Clinical and routine hematological examination was unremarkable except for reduced breath sounds in left lower lobe. Imaging studies were advised. [Figures 1–3] Fine needle aspiration cytology was performed thereafter. [Figure 4] It revealed spindle shaped cells with pleomorphic nucleus and vaiable cytoplasm. (MGG stain, ×100)

**Figure 1a:**
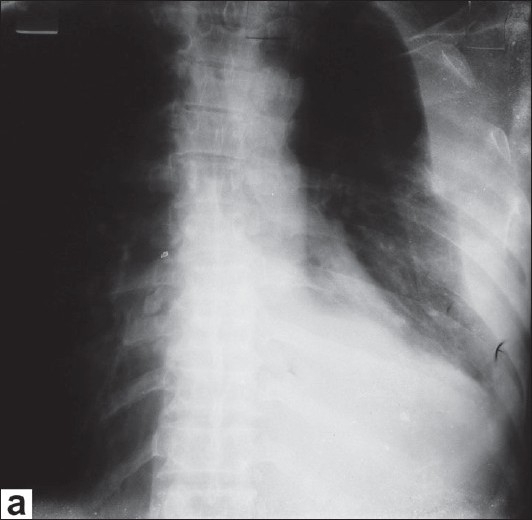
Radiograph chest posteroanterior view

**Figure 1b:**
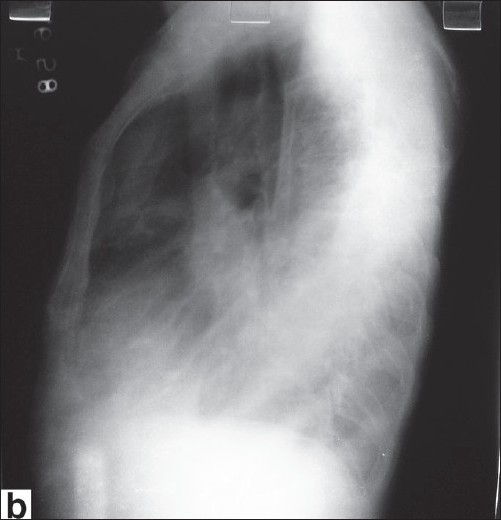
Radiograph chest left lateral view

**Figure 2:**
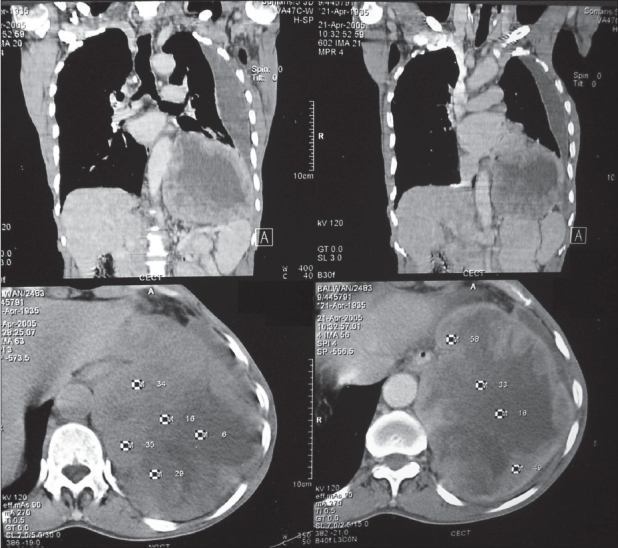
Non contrast and contrast enhanced CT scan images in axial orientation and coronal reformatted images

**Figure 3:**
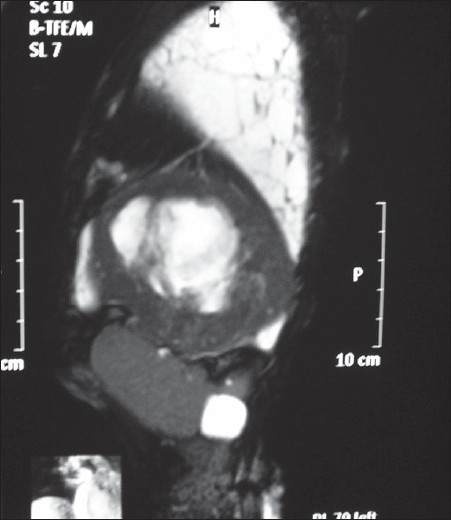
T2-weighted sagittal MRI image

**Figure 4:**
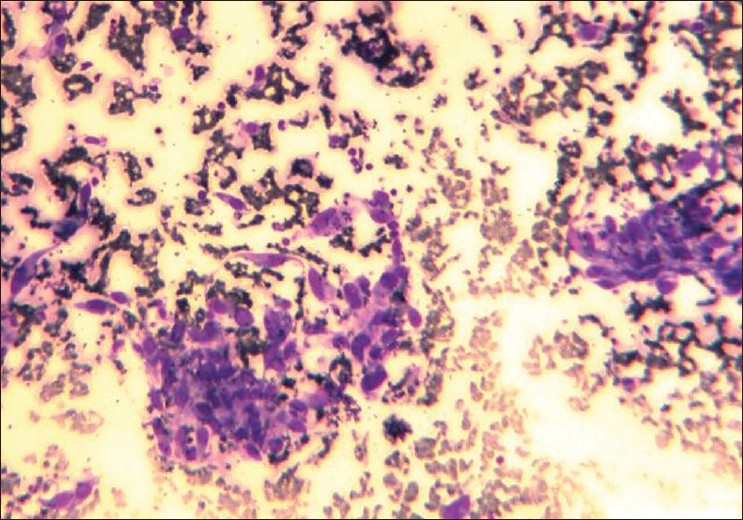
Fine needle aspiration cytology image. (MGG stain, ×100)

## Clinical Questions

1. What are the findings on chest radiographs [Figure 1]?
2. What are the findings on computed tomogram (CT) [Figure 2] and magnetic resonance imaging (MRI) [Figure 3] of the chest? How is MRI useful?
3. What is the differential diagnosis?

## Answers

1. Radiograph chest postero-anterior and lateral views reveal a large homogenous mass of soft tissue density in the left lower zone posterior to and inferior to the cardiac shadow. There is also left sided pleural effusion.
2. Computed tomographic images in the axial orientation and reconstructed coronal images reveal a large heterogeneously enhancing mass lesion with central necrosis along the left hemidiaphragm displacing the spleen and lower lung fields. On MR imaging the lesion is hypointense on both T1-weighted and T2-weighted images with central area of necrosis. In addition there was a loculated left sided effusion. Magnetic Resonance Imaging with its better soft tissue delineation is able to define the structure of origin better which is the diaphragm in this case.
3. Differential diagnosis includes retroperitoneal tumors like leiomyosarcoma and malignant fibrous histiocytoma and pleural tumors like fibroma and mesothelioma with secondary involvement of the diaphragm.

## Diagnosis

Leiomyosarcoma of the diaphragm.

## Discussion

Primary neoplasms of the diaphragm are extremely rare and their diagnosis is often difficult. Most patients present with nonspecific symptoms like chest wall or abdominal pain, cough, and dyspnea and 20% of the patients are discovered incidentally on routine chest X-rays. Arthralgias and hypertrophic pulmonary osteoarthropathy presenting as clubbing are rare manifestations, which may resolve following excision of the tumor.[1,2]

Grancher was the first to discover the primary tumor of the diaphragm during an autopsy study.[3] Wiener and Chou[1] reviewed 71 cases of primary tumors of the diaphragm reported from 1886 to 1963. In 1971, Olafsson *et al*.[4] described 14 cases of primary diaphragmatic tumors reported from 1964 to 1968, and between 1969 and 1995, 55 such cases were reported in the literature.[5] There have been less than 200 cases described in the English Literature between 1868 and 2005.[6] Thereafter only 13 case reports of primary diaphragmatic tumors could be found in the literature.

Patients with diaphragmatic tumors often present between 40 and 60 years of age. Men and women are affected equally. Malignant lesions outnumber benign and the ratio of malignant to benign tumors as reported in the literature is 3:2.[5] Diphragmatic tumors may be divided into the following groups: (1) Primary benign neoplasms; (2) Primary malignant neoplasms; (3) secondary malignant neoplasms; (4) cysts; (5) inflammatory lesions; and (6) endometriosis.[7] The former includes Lipoma (the most common), Angiofibroma, Neurofibroma, etc. Malignant neoplasms include Fibrosarcoma (the most common), Malignant fibrous histiocytoma, Leiomyosarcoma, etc. Various non-neoplastic abnormalities that form localized tumors such as Lymphangioma and Endometrioma are also occasionally found.

Leiomyosarcoma develops relatively frequently in the uterus and digestive tract, and somewhat less frequently in the skin, ovary, retroperitoneum and mesentry. On the other hand, primary leiomyosarcoma of the diaphragm is extremely rare. It was first discovered by Kirschbaum in 1935.[8] Only approximately 10 cases including ours have been reported since 1935.[9] Due to lack of symptoms, and unresponsiveness to various therapeutic modalities, the prognosis of leiomyosarcoma is very poor.[8]

Diphragmatic tumors are often found on chest radiograph first, which reveals, however, non-specific findings such as deformation or elevation of diaphragmatic shadow.[7] Ultrasonography (US), CT and MRI are useful. In particular, MRI is useful in localizing the tumor and determining the relationship with other organs. Diagnostic criteria for the qualitative diagnosis of a diaphragm tumor have not yet been established due to the small number of patients. The only qualitative diagnostic method is biopsy, although it is often difficult.[8]

The treatment of choice for diaphragmatic tumors is surgical excision with thoracotomy providing optimal exposure. However, laparatomy with subcostal incision has been reported as an alternative for surgical approach of the diaphragmatic tumors.[2]
